# Irregular transcriptome reprogramming probably causes thec developmental failure of embryos produced by interspecies somatic cell nuclear transfer between the Przewalski’s gazelle and the bovine

**DOI:** 10.1186/1471-2164-15-1113

**Published:** 2014-12-16

**Authors:** Yongchun Zuo, Yu Gao, Guanghua Su, Chunling Bai, Zhuying Wei, Kun Liu, Qianzhong Li, Shorgan Bou, Guangpeng Li

**Affiliations:** The Key Laboratory of National Education Ministry for Mammalian Reproductive Biology and Biotechnology, Key Laboratory of Herbivore Reproductive Biotechnology and Breeding Ministry of Agriculture, Inner Mongolia University, Hohhot, 010070 China; Laboratory of Theoretical Biophysics, School of Physical Science and Technology, Inner Mongolia University, Hohhot, 010021 China; The Key Laboratory of National Education Ministry for Mammalian Reproductive Biology and Biotechnology, Inner Mongolia University, Hohhot, 010070 China

**Keywords:** Interspecies somatic cell nuclear transfer (iSCNT), Transcriptome reprogramming, Embryonic genome activation, Transcriptional regulation, Mitochondrial DNA

## Abstract

**Background:**

Interspecies somatic cell nuclear transfer (iSCNT) has been regarded as a potential alternative for rescuing highly endangered species and can be used as a model for studying nuclear–cytoplasmic interactions. However, iSCNT embryos often fail to produce viable offspring. The alterations in normal molecular mechanisms contributing to extremely poor development are for the most part unknown.

**Results:**

Przewalski’s gazelle–bovine iSCNT embryos (PBNT) were produced by transferring Przewalski’s gazelle fibroblast nuclei into enucleated bovine oocytes. The percentages of PBNT embryos that developed to morula/blastocyst stages were extremely low even with the use of various treatments that included different SCNT protocols and treatment of embryos with small molecules. Transcriptional microarray analyses of the cloned embryos showed that the upregulation of reprogramming-associated genes in bovine–bovine SCNT (BBNT) embryos was significantly higher than those observed in PBNT embryos (1527:643). In all, 139 transcripts related to various transcription regulation factors (TFs) were unsuccessfully activated in the iSCNT embryos. Maternal degradation profiles showed that 1515 genes were uniquely downregulated in the BBNT embryos, while 343 genes were downregulated in the PBNT embryos. Incompatibilities between mitochondrial DNA (mtDNA) and nuclear DNA revealed that the TOMM (translocase of outer mitochondrial membrane)/TIMM (translocase of inner mitochondrial membrane) complex-associated genes in BBNT embryos had the highest expression levels, while the PBNT embryos exhibited much lower expression rates.

**Conclusions:**

Improper degradation of maternal transcripts, incomplete activation of TFs and abnormal expression of genes associated with mitochondrial function in PBNT embryos likely contributed to incomplete reprogramming of the donor cell nuclei and therefore led to the developmental failure of these cloned embryos.

**Electronic supplementary material:**

The online version of this article (doi:10.1186/1471-2164-15-1113) contains supplementary material, which is available to authorized users.

## Background

Przewalski’s gazelle *(Procapra przewalskii)* is one of the more critically endangered Eurasian large mammals and is unique to China. Only around 350–400 mature individuals are thought to remain [1]. Their range exists today only in a small area surrounding Qinghai Lake [2, 3]. This gazelle’s fate is considered to be even more precarious than the giant panda [4]. Rescue and conservation programs are a challenge for wildlife biologists and ecologists, although management efforts are underway to provide for a more sustainable population [5]. Somatic cell nuclear transfer (SCNT) has been successfully utilized in the production of many mammal species including laboratory and domestic animals. One potential application of this technology is that it might be useful for the propagation of rare and endangered species. However, the major limitation in using this technology for species rescue is that oocytes and suitable recipients are rare, so intraspecies cloning of endangered species becomes an even more daunting task. Interspecies SCNT (iSCNT) where endangered animal somatic cell nuclei are transferred to domestic oocyte cytoplasts is an approach that might minimize the limitations of SCNT. Many trials of iSCNT have been reported in wildlife species such as the giant panda (*Ailuropoda melanoleuca*) [6, 7], Tibetan antelope (*Pantholops hodgsonii*) [8], Banteng (*Bos javanicus*) [9], yak (*Bos grunniens*) [10], Siberian tiger (*Panthera tigris altaica*) [11], and Sei whale (*Balaenoptera borealis*) [12]. The iSCNT cloned embryos generated in these studies had extremely poor development to the blastocyst stage. The best results of iSCNT in mammals occurred when using subspecies and sibling species that can hybridize naturally, such as among the cloned argali (*Ovis ammon*) [13] and the river buffalo (*Bubalus bubalus arnee*) [14].

The current hypotheses for the high developmental failure of iSCNT are that there is genomic incompatibility between the nucleus and the host ooplasm/cytoplasm [15] and between mitochondrial DNA (mtDNA) and nuclear DNA [16]. A major barrier that hinders the developing iSCNT embryo first occurs at the time of zygotic genome activation (ZGA) or embryonic genome activation (EGA) [17]. The zygote genome of the cloned embryo manifests itself independently from the maternal transcripts [18]. Compared with the development of normally fertilized preimplantation embryos, SCNT-derived embryos have to overcome many more challenges in silencing their somatic-specific genes while reactivating all of the embryo-related genes [19]. During the process, it also has to shed its differentiated phenotype and establish a totipotent state [20]. At the present time there are very few reports on embryonic gene function at the time of EGA in iSCNT embryos. Although it has been shown that small molecules such as valproic acid (VPA) [21], trichostatin A (TSA) [22], 5-aza-2′-deoxycytidine [23] can improve SCNT embryo development *in vitro* and *in vivo*, such beneficial effects have not been observed in interspecies cloned embryo development.

We transferred Przewalski’s gazelle fibroblast nuclei into enucleated bovine oocytes in this study. The Przewalski’s gazelle–bovine nuclear transfer (PBNT) embryos were treated with VPA and TSA and monitored for embryo development. However, the trials did not result in a significant improvements in iSCNT embryo development. To better understand why the embryos failed to develop and thrive, we used genomic computational methods to analyze the global reprogramming transcriptome between PBNT embryos and bovine–bovine nuclear transfer (BBNT) embryos at the time of the maternal–zygotic genomic transition (MZT). We first identified and then systematically analyzed the different gene expression patterns between PBNT and BBNT embryos in the global transcriptome, maternal mRNA degradation, transcription regulation-related genes and the aberrant expression of genes associated with mtDNA. Result of the quantitative PCR in the SCNT embryo and in the iSCNT embryos revealed excellent agreement with the microarray data. Putative mechanisms affecting developmental potential between SCNT and iSCNT embryos are discussed.

## Results

### Treatment of PBNT embryos with VPA did not affect embryo development

Fused PBNT embryos were treated with VPA at 0.5, 1.0, 2.0 and 4.0 mM for 24 h. Two- and 8–16 cell embryo development rates were significantly higher in the 0.1 and 1.0 mM and control groups than in the 2.0 and 4.0 mM VPA groups (Table 1). There were no beneficial effects on morula or blastocyst development rates among VPA treatments and the controls. Fewer cloned embryos developed to blastocysts in the 0.5 mM (0.7%), 1.0 mM (0.7%) and control groups (1.5%). Better results were obtained in the 0.5 mM VPA group. When the embryos were treated with VPA at 0.5 mM for 24 h, the initial cleavage and 8–16-cell developmental rates were significantly better than in treatment times of 5, 12 and 48 h (Table 1). This improvement in development was not observed in the morula and blastocyst stage. There was no difference among the treatments and the controls. Longer exposure times to VPA did not improve PBNT embryo development.

**Table 1:** **PBNT embryo development after VPA treatment**

Treatment		No.embryos cultured	Cleavage (%)	8-16 cells (%)	Morula (%)	Blastocysts (%)
VPA at different concentrations (mM)	Control	136	98(72.1)^a^	69(50.7)^a^	2(1.5)	2(1.5)
	0.5	153	120(78.4)^a^	95(61.9)^a^	2(1.3)	1(0.7)
	1.0	147	104(70.7)^a^	75(51.0)^a^	1(0.7)	1(0.7)
	2.0	138	81 (58.7)^b^	29(21.0)^b^	1(0.7)	0(0.0)
	4.0	158	79(50.0)^b^	12(7.6)^b^	0(0.0)	0(0.0)
VPA at 0.5 mM for differenttimes (h)	Control	118	83(70.3)^a^	52(44.1)^b^	1(0.8)	1(0.8)
	5	142	99 (69.7)^a^	60(42.3)^b^	1(0.7)	1(0.7)
	12	151	107(70.9)^a^	71(47.0)^b^	1(0.7)	1(0.7)
	24	146	111(76.0)^a^	95(65.1)^a^	1(0.7)	1(0.7)
	48	149	102(68.5)^a^	74(49.7)^b^	0(0.0)	0(0.0)

In the column, the same letter in superscript denotes no significant difference, different letter in superscript denotes significant difference. p<0.05.

The PBNT embryos derived from green fluorescent protein (GFP)-expressing Oct-4-eGFP transgenic cells were further treated with 0.5 mM VPA for 24 h. Cleavage rates, 8–16-cell, morula and blastocyst development rates were no different between treatment groups and controls (Additional file 1: Table S1). Treatment of Oct-4-eGFP-derived PBNT embryos with VPA had no effect on embryo development.

### Reverse nuclear transfer (RNT) did not improve PBNT embryo development

The reverse nuclear transfer (RNT) method significantly improved the blastocyst development rate in BBNT (36% in RNT *vs* 26% in SCNT). Whereas RNT did not improve the rate of PBNT embryo development (Table 2).

**Table 2:** **Development of PBNT embryos derived from the reverse nuclear transfer protocol**

Intra/inter	NT protocol	No.embryos cultured	Cleavage (%)	8-16 cells (%)	Morula (%)	Blastocysts (%)
P-B	NT	90	55(61.1%)^b^	41(45.6%)^b^	1(1.1%)	0
P-B	RNT	96	61(63.5%)^b^	47(49.0%)^b^	1(1.0%)	1(1.0%)
B-B	NT	109	79(72.4%)^a^	68(62.4%)^a^	33(30.3%)^b^	29(26.6%)^b^
B-B	RNT	75	53(70.7%)^a^	49(65.3%)^a^	30(40.0%)^a^	27(36.0%)^a^

In the column, the same letter in superscript denotes no significant difference, different letter in superscript denotes significant difference. p<0.05.

### Scatterplot comparison of different microarray datasets

**Figure 1:**
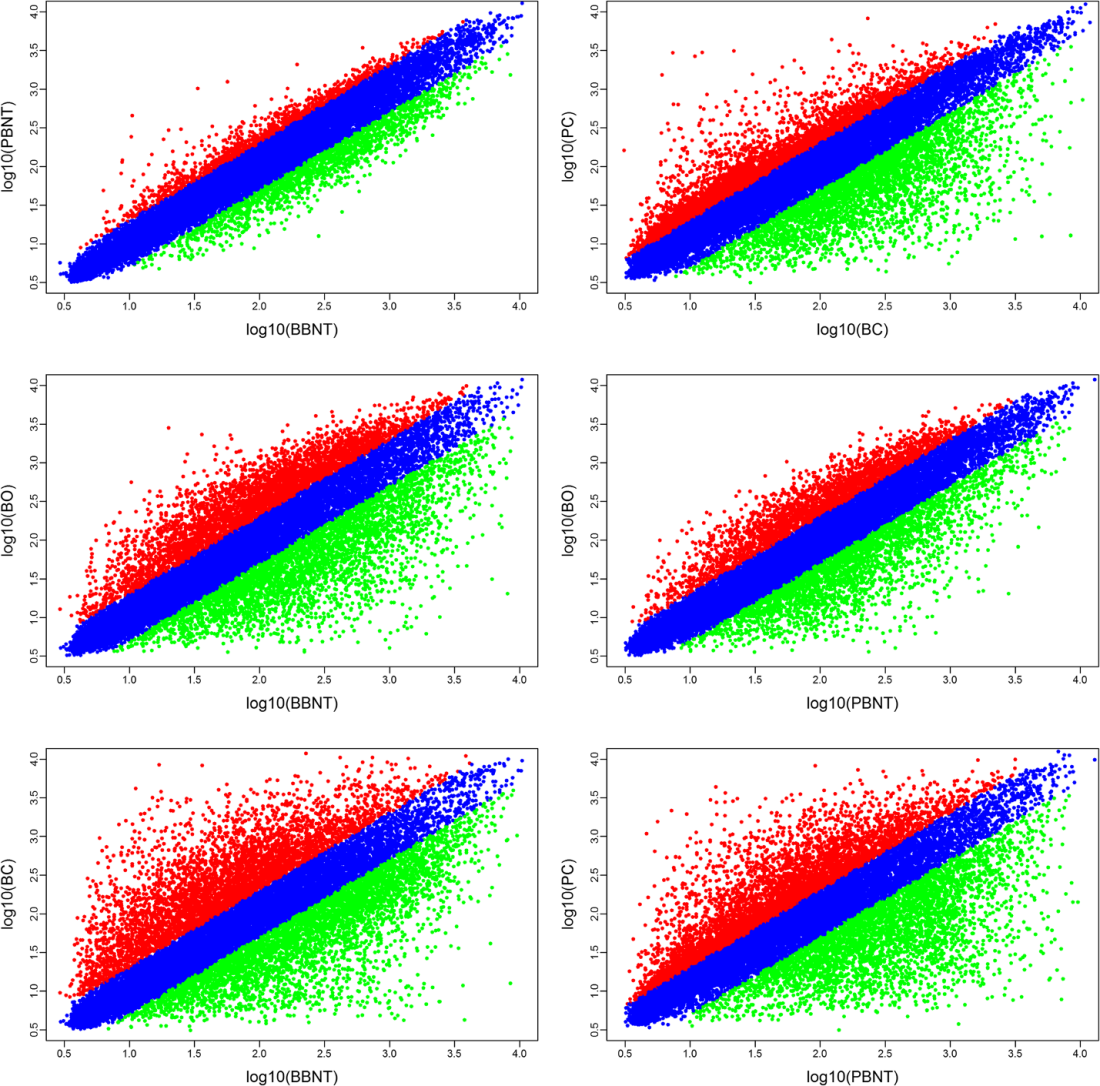
**Scatter plot compares the results of log transformed gene expression levels and the differentially expressed gene distribution pattern between the two cell types.** Green indicates down- and red up-regulation of gene expression.

Gene array analysis was performed using the Affymetrix gene chip bovine genome array (Santa Clara, CA, USA). A total of 1150 bovine oocytes (BOs), 309 8- to 16-cell BBNT embryos, 527 8- to 16-cell PBNT embryos, Przewalski’s gazelle fibroblasts (PCs) and bovine fibroblasts (BCs) were used in the computational analyses. The developmental stage and the morphology of the iSCNT embryos were with no obvious different from the control intra-species NT embryos (Additional file 2: Figure S1). High reproducibility was obtained between the replicates and datasets. The scatterplot compared the results of the log transformed gene expression levels and the differentially expressed gene profiles between two cell types (Figure 1). All of the treatments were repeated at least three times. High reproducibility was obtained between the replicates and datasets. The scatter plot compared the results of the log transformed gene expression levels and the differentially expressed gene profiles between two cell types (Figure 1). The Spearman correlation coefficients between the profiles of different cell types reflect the degree of change in transcriptomes (Additional file 3: Table S2). The results showed the BBNT oocytes and PBNT embryos had the most similar transcriptional profiles (R^2^ = 0.97) than other comparisons.

### Reprogramming transcriptome analysis of BBNT and PBNT embryos

**Figure 2:**
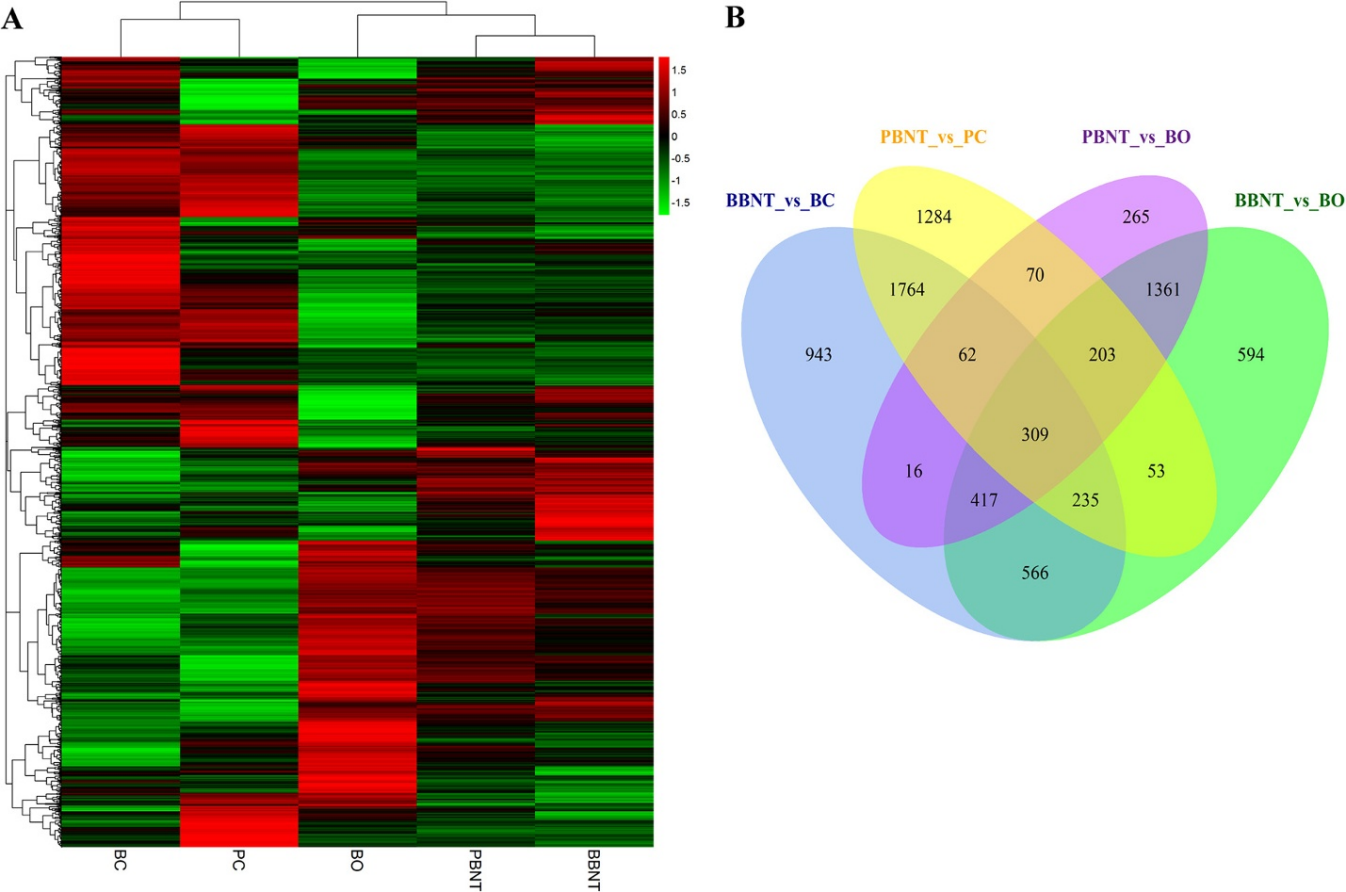
**The hierarchical analyses and shared co-expression transcripts for different cell types. (A)**. Hierarchical clusters of overall gene expression profiles. Green indicates the down-and red the up-regulated gene expression. **(B)**. Venn diagram of shared and unique genes among different transcriptomes.

Hierarchical clustering of complete transcriptional profiling between the two different chip platforms indicated separations between any two sample groups (Figure 2A). More than 10,000 transcripts were detected in each sample, with similar profile patterns clustered between any two close cell or embryo types based on average Euclidean distance. The BCs and PCs showed highly unique and consistent expression patterns. The clustered coherent profiles revealed that BBNT embryos had more upregulated genes than did PBNT embryos at the same development stages (Additional file 4: Table S3). A Venn diagram illustrating shared and unique genes between BBNT and PBNT embryos, PCs and BCs, bovine oocytes and various regulatory factors is illustrated in Figure 2B.

**Figure 3:**
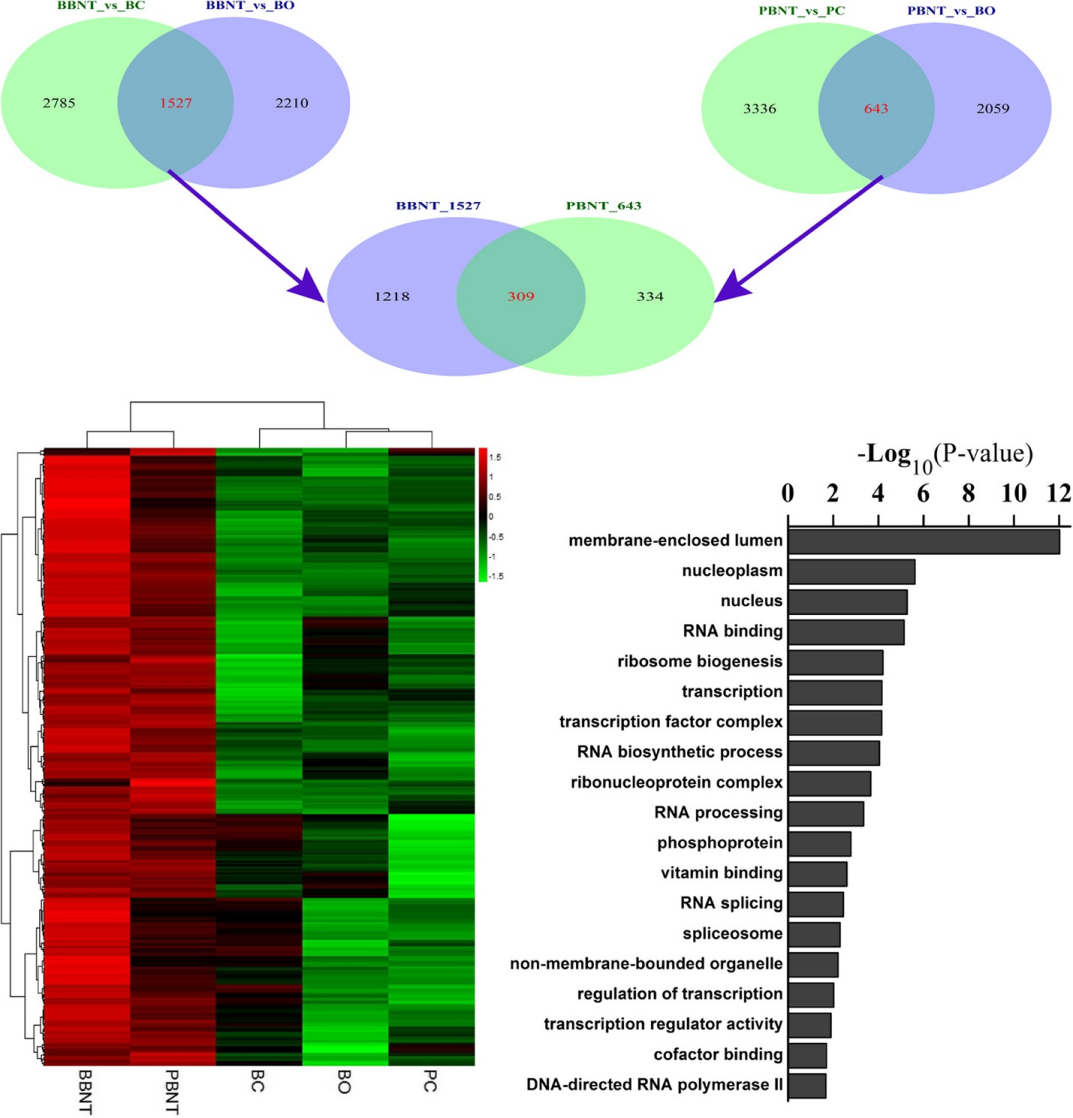
**The co-expression of reprogramming genes and the functional classification categories between BBNT and PBNT embryos.** Green indicates down-and red up-regulation of gene expression.

Upon subtracting the genes expressed in the bovine oocytes and in the PCs and BCs from the transcriptome, there were, 1527 and 643 upregulated genes associated with nuclear reprogramming in the BBNT and PBNT embryos, respectively (Venn diagrams in Figure 3; Additional file 5: Figure S2 and Additional file 6: Table S4), which were 2.4 times higher in BBNT than in PBNT embryos. Three hundred nine reprogramming related genes were identified as being co-expressed in both the BBNT embryo and PBNT embryo samples. These co-expressed genes are associated with several important biological processes such as membrane-enclosed lumen, RNA processing/splicing, RNA biosynthetic processing and transcriptional regulation (Figure 3, Additional file 7: Table S5). These results were consistent with the recently published studies of single-cell RNA sequencing analysis [24, 25].

The Venn diagrams in Figure 3 show that there were 1218 uniquely upregulated genes in BBNT embryos and 334 upregulated genes in PBNT embryos. The heatmap profile showed that these two gene clusters are significantly diverse (Additional file 8: Figure S3). In the top five upregulated gene clusters, the most significant biological processes in Table 3 were related to nuclear composition, ribosome biogenesis, tRNA/rRNA metabolic processes and mRNA splicing. Significant differences between the mRNA expression profiles were related to transcriptional regulation. It has been demonstrated that transcription regulation is critical for early transcriptional activation during the MZT [26].

**Table 3:** **Functional annotation of specific expressed transcripts between BBNT and PBNT embryos**

ID	BBNT		PBNT	
**Top-1**	**Enrichment Score:19.96**	**P-Value**	**Enrichment Score:6.52**	**P-Value**
	nuclear lumen	3.86E-34	nuclear lumen	5.87E-11
	intracellular organelle lumen	9.19E-30	membrane-enclosed lumen	1.20E-09
	organelle lumen	1.07E-29	intracellular organelle lumen	2.79E-09
	membrane-enclosed lumen	1.78E-29	organelle lumen	2.95E-09
	Nucleolus	2.14E-26	nucleolus	7.55E-09
	nucleoplasm part	1.23E-10	nucleoplasm part	0.001186
	nucleoplasm	4.58E-10	nucleoplasm	0.001195
	intracellular non-membrane-bounded organelle	5.13E-08	nucleus	0.010386
**Top-2**	**Enrichment Score:7.05**	**P-Value**	**Enrichment Score: 1.85**	**P-Value**
	ribosome biogenesis	9.45E-13	ribosome biogenesis	2.55E-04
	ribonucleoprotein complex biogenesis	2.17E-12	ribonucleoprotein complex biogenesis	0.001333
	ribosome biogenesis	4.54E-08	ncRNA metabolic process	0.009469
	ncRNA processing	5.01E-07	ncRNA processing	0.01394
	rRNA metabolic process	3.52E-06	rRNA metabolic process	0.063108
	rRNA processing	3.52E-06	rRNA processing	0.063108
	ncRNA metabolic process	1.63E-05	tRNA metabolic process	0.124026
	rrna processing	3.97E-04	tRNA processing	0.190786
**Top-3**	**Enrichment Score: 6.91**	**P-Value**	**Enrichment Score:4.72**	**P-Value**
	**transcription**	**9.72E-13**	intracellular non-membrane-bounded organelle	9.51E-04
	**regulation of transcription**	**2.27E-06**	non-membrane-bounded organelle	9.51E-04
**Top-4**	**Enrichment Score: 6.35**	P-Value	**Enrichment Score: 1.61**	**P-Value**
	RNA processing	4.62E-17	RNA processing	9.21E-05
	RNA splicing	2.90E-08	mRNA processing	0.011515
	mRNA processing	4.54E-08	Spliceosome	0.012021
	mRNA metabolic process	4.72E-08	RNA splicing	0.014729
	mrna splicing	7.03E-06	mRNA metabolic process	0.025064
	Spliceosome	5.85E-04	mrna splicing	0.053721
	ribonucleoprotein complex	0.005378		
**Top-5**	**Enrichment Score: 4.33**	**P-Value**	**Enrichment Score:2.23**	**P-Value**
	**transcription DNA-dependent**	**1.76E-05**	vitamin binding	1.44E-04
	**RNA biosynthetic process**	**2.96E-05**	transaminase activity	2.62E-04
	**transcription from RNA polymerase II promoter**	**1.93E-04**	Aminotransferase	6.60E-04
			transferase activity, transferring nitrogenous groups	0.001604
			cofactor binding	0.005546

**Figure 4:**
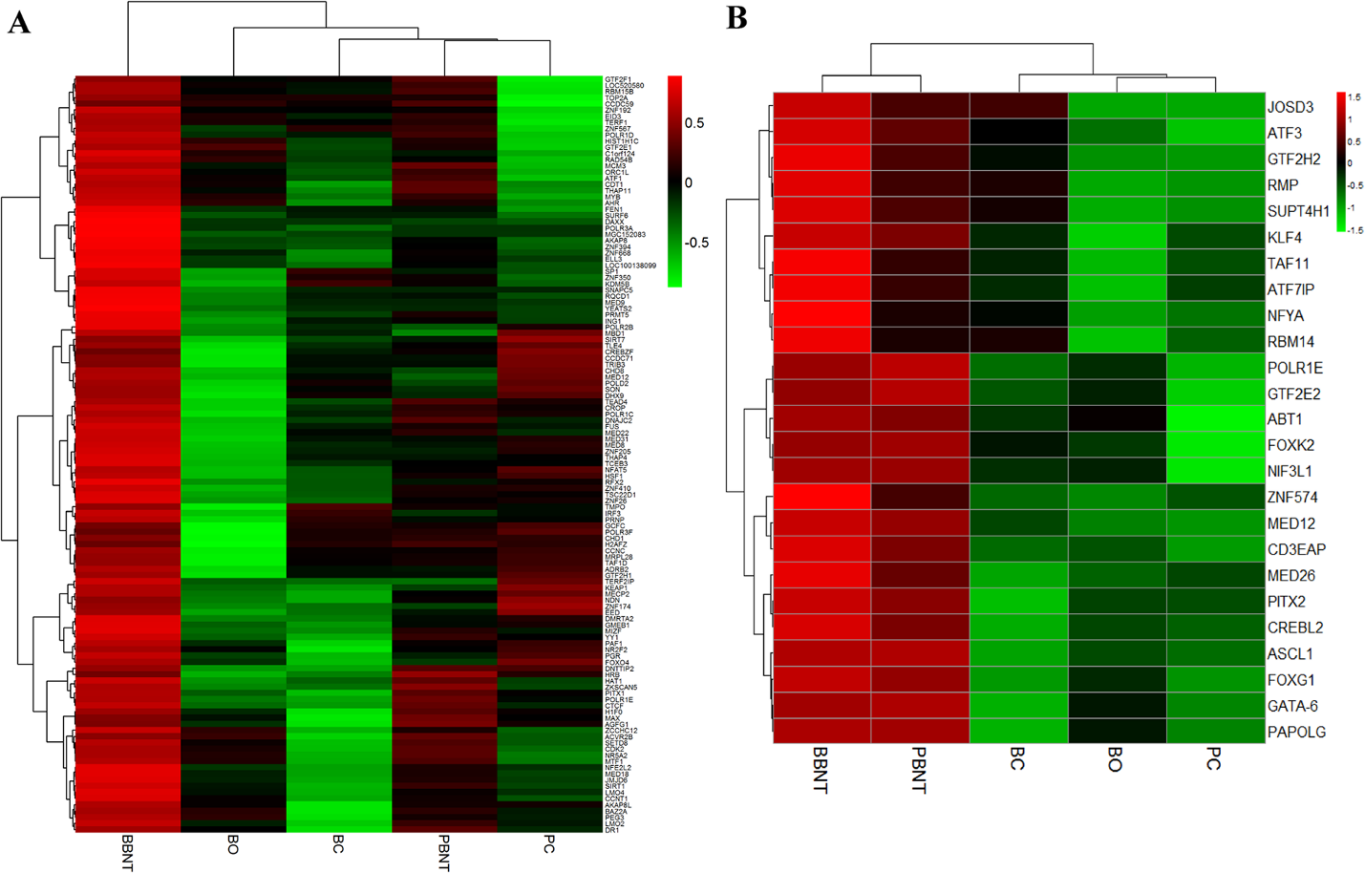
**Expression clusters of transcriptional regulated related genes. (A)**. The 139 unique upregulated transcripts in BBNT embryos that associated with transcriptional regulation. **(B)**. The 25 co-upregulated transcripts of transcription regulation in both BBNT and PBNT embryos. Green represents down- and red up-regulation of gene expression.

There were 25 of the transcription regulator-associated genes co-upregulated in both BBNT and PBNT embryos (Figure 4B). One hundred thirty-nine transcripts associated with transcriptional regulation were upregulated in BBNT embryos, whereas these genes had only low expression levels in PBNT embryos (Figure 4A; Additional file 9: Table S6). These genes contribute to multiple biological functions such as general transcription factors, mediator complexes, nuclear receptor subfamily kinase, anchor proteins, RNA polymerases, and zinc finger proteins. As an example, the basic transcription factors for RNA polymerase (e.g., TBP, TFIIB, TAF1D, SP1 and TAF2) were all upregulated in BBNT embryos, but only TBP and TAF2 were upregulated in PBNT embryos (Additional file 10: Figure S4). Unsupervised hierarchical clustering revealed that the expression pattern of these transcription factors in PBNT embryos were more similar to the donor somatic cells, which indicates that the key transcription regulatory pathway in PBNT embryos did not activate as in BBNT embryos (Figure 3).

**Figure 5:**
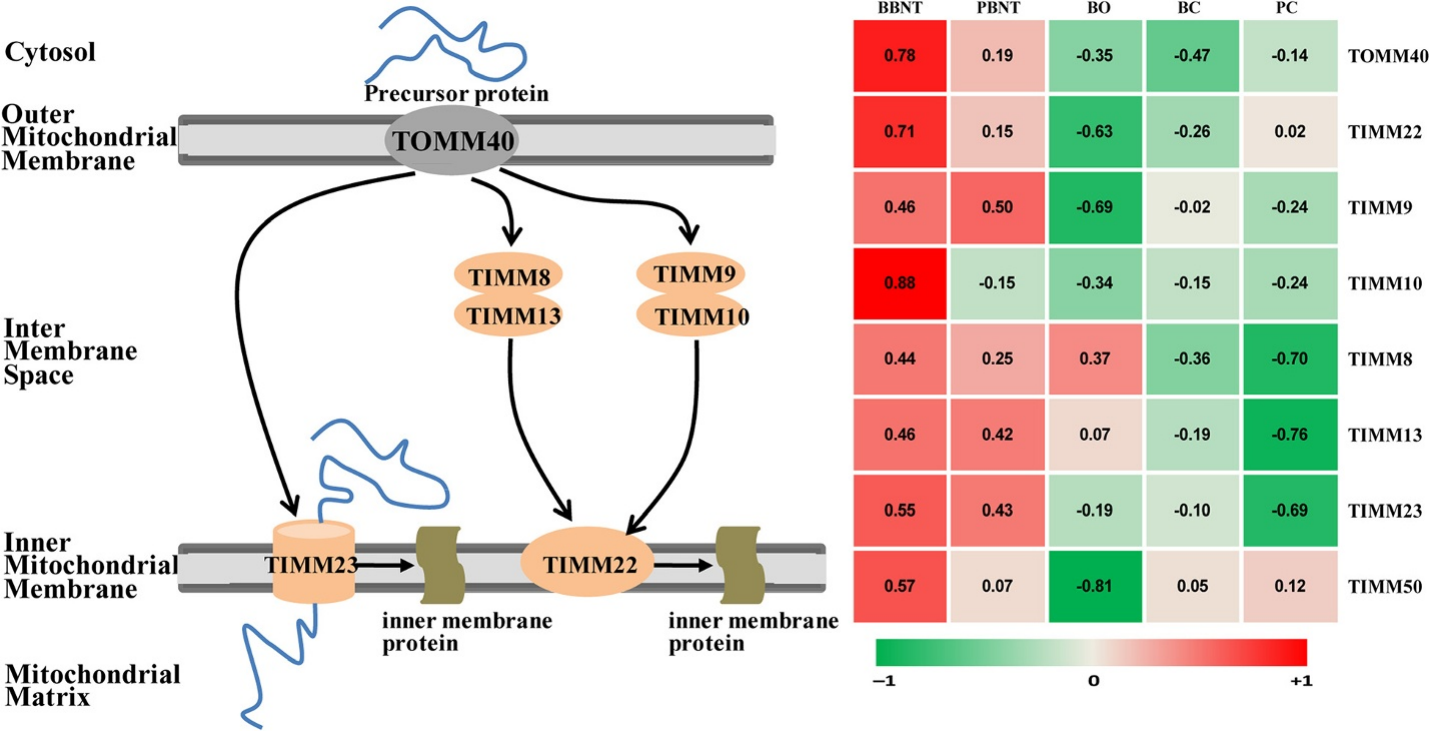
**Cooperation of translocase complexes in mitochondrial protein import and expression comparisons in different cell types.**

**Figure 6:**
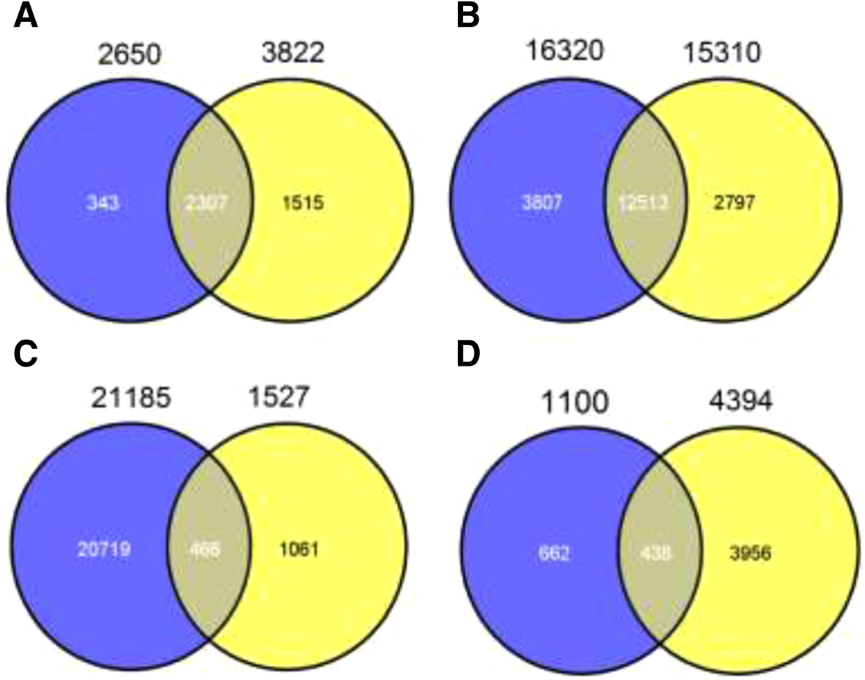
**Venn diagram of differentshared and uniquetranscripts in PBNT and BBNT embryos. A**. Venn diagrams showing the same down-regulated genes between iSCNT embryosvs bovine oocytesand SCNT embryosvs bovine oocytes (2,307 genes). The blue circle (2,650 genes) indicates the number of down-regulated genes in iSCNT embryosvs the bovine oocytes, and theyellow circle (3,822 genes)indicates the number of down-regulated genes in the SCNT embryosvs the bovine oocytes. **B**. Venn diagram showing the same non-regulated genes between iSCNT embryosvs Przewalski’s gazelle fibroblasts and SCNT embryosvs bovine fibroblasts (12,513 genes). The blue circle (16,320 genes) indicates the number of non-regulated genes in the iSCNT embryosvs the Przewalski’s gazelle fibroblasts, andyellow circle (15,310 genes)indicates the number of non-regulated genes in the SCNT embryosvs bovine fibroblasts. **C**. Venn diagram showing common gene expression between iSCNT and SCNT embryos (466 genes). The blue circle denotes transcripts that have similar expression levels between iSCNT embryos vs SCNT embryos (21,185 genes), andthe yellow circle indicates reprogrammed transcriptomes in SCNT embryos (1,524 genes). **D**. Venn diagram showing somatic gene expression in 8- to 16-cell stage iSCNT embryos (438 genes) compared toSCNT embryos. The blue circle denotes up-regulated genes in iSCNT compared to SCNT embryos (1,100), and yellow circle denotes up-regulated genes in bovine fibroblasts compared to SCNT embryos (4,394).

Incompatibility between mtDNA and nuclear DNA is regarded as another one of the major problems impairing iSCNT embryo development. Most mitochondrial proteins are encoded in the nucleus, and thus have to be transported into one of the four sub-compartments of the organelle (Figure 5). These processes are mainly mediated by a general translocase in the outer mitochondrial membrane (TOMM: TOMM40), and two distinct translocases in the mitochondrial inner mitochondrial membrane (TIMM: TIMM23 and TIMM22 complexes) [27]. For example, TOMM40 is the channel-forming subunit of the TOMM complex and has an essential role for protein import into the mitochondria. TIMM proteins mediate the import and insertion of hydrophobic membrane proteins into the mitochondrial inner membrane. In this study, the gene transcripts related to the TOMM/TIMM complex in BBNT embryos had the highest expression when compared with the BOs and fibroblasts. Most of the mitochondrial protein import genes in the PBNT embryos such as TOMM40, TIMM10, and TIMM50, had lower expression levels than in the BBNT embryos.Ninety-seven percent of the PBNT embryos did not develop beyond the 8- to 16-cell stage, which is regarded as the crucial time for MZT. The maternal degradation profiles showed that 3822 genes in BBNT embryos and 2650 genes in PBNT embryos were downregulated. There were 2307 genes shared between BBNT and PBNT embryos. Of these genes, 1515 were uniquely downregulated in the BBNT embryos, whereas 343 genes were downregulated in PBNT embryos (Figure 6A). These results suggest that the BBNT embryos were capable of eliciting a more significant degradation of maternal RNA than the PBNT embryos.

Theoretically, only donor cell nuclei that completely reprogram in the host cytoplast have a reasonable probability of developing to term following SCNT. After analysis of the donor-specific genes, we found there were more donor-specific transcripts expressed in PBNT embryos than in BBNT embryos (16,320 *vs* 15,310). The two kinds of embryos shared 12,513 transcripts (Figure 6B). We also observed 438 abnormal fibroblast-specific genes expressed in the PBNT embryos (Figure 6D). The host cytoplast did not silence enough of the donor-specific genes, which is consistent with other reports [28, 29]. For example, the collagen-related protein, Col4A1, was upregulated 2.2 times higher in PBNT than in BBNT embryos. There were 21,855 nonsignificant discrepancy transcripts shared between the two kinds of cloned embryos. When we compared these genes with the SCNT embryonic reprogrammed transcriptome, 466 genes were shared in both datasets (Figure 6C).

### Quantitative PCR confirmation of array data

**Figure 7:**
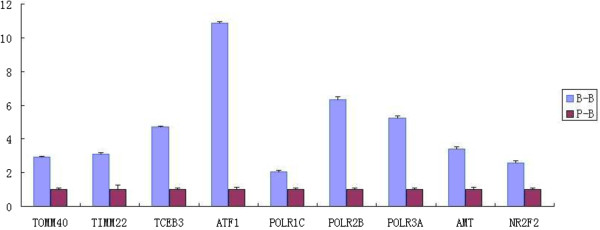
**Quantification of relative abundance of nine transcripts between the 8–16 cell stage of the embryos (72h post activation).** Przewalski’s gazelle-bovine interspecies SCNT embryos (P-B) and bovine intra-species SCNT embryos(B-B) in expression of nine select genes as determined by quantitative PCR. Data are presented as mean value of triplicate measurements including standard errors.

For the validation of microarray datasets results, nine genes showing high levels of significance (TOMM40, TIMM22, TCEB3, ATF1, POLR1C, POLR2B, POLR3A, AMT and NR2F2 were selected and their expression were determined by quantitative PCR (Figure 7). Two polymerase (RNA) II polypeptide genes, POLR2B and POLR3A, showed higher expression in the SCNT embryos with an average of 6.3- and 5.2- fold differences, respectively, when measured with qPCR and 2.8- and 3.2-fold differences, respectively, using microarray measure. The other two genes of mitochondrial translocase, TOMM40 and TIMM22, showed an average of 2.9- and 3.1-fold differences, respectively using qPCR and 2.0- and 1.9-fold difference, respectively, with microarray measure. The activating transcription factor 1 (ATF1) showed highest expression in the SCNT embryos with the average of 10.9- fold difference. Thus, all of the evaluated genes showed similar patterns of mRNA abundance in microarray analysis and qPCR measure.

## Discussion

Various studies have shown that the majority of cloned mammalian embryos fail to development because of incomplete genomic reprogramming [30]. Theoretically, the iSCNT embryos derived from inter-class, inter-order or inter-family donor cells and recipient oocytes should result in much more incomplete nuclear reprogramming than that of intra-species cloning because of greater genetic divergence. To assist in the relaxation of chromatin structure and provide for better cell nuclear reprogramming, histone-deacetylase inhibitors and DNA methyl-transferase have been used in SCNT protocols. TSA, VPA and 5-aza-2′-deoxycytidine have been reported to increase cloned blastocyst development rates significantly [21, 31–33]. However treatments with these small molecules do not enhance full-term in vivo development [34]. In iSCNT studies, the use of TSA has not been shown to have any beneficial effect on cloned embryo development in the gaur-bovine [35], human-rabbit [36], and dog-porcine [37]. In our study, the PBNT morula/blastocyst development also did not improve when using TSA, VPA, or Oct 4-transgenic treatments. The coexistence of the donor cell nucleus and oocyte nucleus using the RNT protocol has been reported to facilitate donor cell reprogramming and improve cloned embryo development in mouse [38] and bovine [39]. In our study, PBNT hybrid embryo development was not improved with the RNT protocol.

Genomic incompatibility between the nuclear donor cell and the cytoplast is a major contributing factor causing iSCNT hybrid embryos to arrest at the 8- to 16-cell stage. Wang et al. [17] observed in chimpanzee–bovine iSCNT embryos that the bovine ooplasm partially remodeled the chimpanzee somatic cell nuclei. However, the cloned embryos still arrested at the 8- to 16-cell stages. Lagutina et al. [40] reported that pig–bovine iSCNT embryo development was completely blocked at the 16-cell stage, while the bovine–pig iSCNT embryos stopped earlier at the 4-cell stage. These studies demonstrated that embryo development was completely arrested before ZGA.

Cytoplasmic factors begin the genomic reprogramming process shortly after the somatic cell nucleus is transferred into the oocyte. It is generally agreed that most cloned embryos are not fully activated and that incomplete cellular reprogramming is the major cause of the extremely poor outcome in SCNT and iSCNT experiments. The timing of large-scale activation of the embryonic genome is species-specific. In the bovine embryo it occurs at the 8-cell stage [41]. At the beginning of MZT, the embryo is dependent on a vast store of mRNA and proteins in the recipient cytoplasm. Complete EGA occurs in the later preimplantation development stage. At the MZT stage the embryo should completely reprogram the somatic cell nucleus, silencing the accompanying donor-specific transcripts and then begin its self-sustained cellular directed development [42]. Complete activation of the embryonic genome is required to produce a normal full-term cloned fetus [43]. In inter-specific cloning, the reprogramming process is greatly compromised and becomes an extremely complicated disarray when the two genetically different genotypes are constrained to coexist in the same embryo. We observed 309 genes that showed increased co-expression in the BBNT and PBNT embryos. These normally control important biological processes such as the formation of membrane-enclosed lumens, RNA processing/splicing, RNA biosynthetic processes and transcriptional regulation. There were significant differences in the upregulated genes associated with nuclear reprogramming between PBNT and BBNT embryos. Greater reprogramming occurred in the BBNT embryos than in the PBNT embryos. Our findings are consistent with the most recently published single-cell RNA sequencing analysis, which identified three pathways in human 8-cell stage embryos [24, 25], and they are also consistent with transcriptional activation waves based on a single human embryo study [26].

Communication between the nucleus and cytoplasm is critical for ZGA or EGA, and is essential for normal embryo development [26, 44]. An orderly and complete transition from the maternal to the zygotic genome is crucial for a normal term pregnancy. Irregular or incomplete activation of the embryonic genome will alter the processes required to direct normal embryo development. During MZT, the oocyte’s maternal mRNAs gradually undergo degradation along with gradual activation of the zygotic genes [45]. In this study, maternal degradation profiles differed between PBNT embryos with 2650 genes and BBNT embryos with 3822 genes;, 1515 genes were uniquely downregulated in BBNT embryos whereas there were 343 in PBNT embryos. There were more gazelle cell-specific transcripts detected in PBNT than in BBNT embryos. This observation was consistent with the presence of 438 abnormal fibroblast-specific genes expressed in PBNT embryos. These results suggested that the BBNT embryos were more efficient in degrading maternal RNA than the PBNT embryos, and that the bovine cytoplasts were not very effective in silencing the gazelle cell-specific genes. The inability to silence the reprogramming-associated genes in PBNT embryos might in part arise from various nuclear-cytoplasmic incompatibilities, thus leading to failure of subsequent embryo development [28, 29]. Past studies on SCNT embryos have established a direct relationship between embryonic genome activation and the ability of the embryo to develop [46, 47]. Aberrations in the expression of housekeeping genes and genes dependent on the major embryonic genome activation is a major problem of iSCNT embryos for different species combinations [42]. Human–human, human–bovine, and human–rabbit interspecific clones all had similar rates of development to the 8- to 16-cell stage. Gene expression profiles of human–human embryos had similar levels of upregulation as the control fertilized embryos, which is not the case for interspecies clones [18, 42].

The extremely low developmental rates of PBNT embryos in this study strongly suggest that aberrant gene expression and altered transcriptional regulation are major contributing factors leading to poor embryo development. Even though there were 25 transcription regulators co-upregulated in the BBNT and PBNT embryos, more than 100 genes involved in transcriptional regulation were widely expressed in BBNT embryos but not in the PBNT embryos. The unsupervised hierarchical clustering utilized in this study showed that expression patterns of the transcription factors in PBNT embryos were more closely aligned to the donor somatic cell nucleus, which indicates in all likelihood that the key transcription regulatory pathway of PBNT was not activated as it was in BBNT embryos. In chimpanzee–bovine iSCNT experiment where the transcription factors Oct-4, Stella, Crabp1, CCNE2, CXCL6, PTGER4, H2AFZ, c-MYC, KLF4, and GAPDH were expressed, the chimpanzee nuclei were only partially remodeled and the cloned embryos did not develop beyond the 8- to 16-cell stages [17]. Even when using Oct-4 transgenic cells, blastocyst development was also not improved in our PBNT embryos. It appears that the downregulation of genes associated with transcription and transcription regulation was only partially reprogrammed in PBNT embryos, which was not enough to support normal zygotic genome activation.

Another major obstacle preventing nuclear–cytoplasmic compatibility is the interaction between donor cell nuclear DNA, mtDNA and oocyte mtDNA [48–51]. An iSCNT study has indicated that the donor cell mtDNA gradually becomes dominant in the post-implantation embryo and during fetal development [35]. Mitochondria promote a broad range of critical functions and have to provide sufficient energy for reprogramming and embryonic genome activation during embryonic and fetal development [40, 48]. Mitochondrial impairment or insufficiency has been reported to impair the efficacy of SCNT [52]. Our data revealed that the TOMM/TIMM complex-associated genes in BBNT embryos had significantly higher expression levels than in PBNT embryos. It has been estimated that over 1000 proteins involved in mitochondrial function are encoded by nuclear DNA, synthesized in the cytosol, and then targeted and imported into the mitochondria by specific transfer pathways [52–54]. Any alterations in the mitochondrial protein import pathway resulting from incompatibilities of heterogeneous mtDNA and genome DNA would likely lead to developmental failure of PBNT embryos. Thus, inefficiency in mitochondrial protein importation mechanisms might be a major contributor to iSCNT embryo failure.

## Conclusion

The gene expression network determines the cell’s identity and behavior. The latest transcriptome profiles from single-cell RNA Sequencing provides convincing evidence that the human preimplantation transcriptional organization is highly preserved, that gene activation is sequentially ordered and genetic programming is essential for preimplantation development. Our study provided a comprehensive comparison of intra/inter bovine embryonic transcriptome during preimplantation development. Significant differences were observed in the mRNA expression profiles between the BBNT and PBNT cloned embryos. Comparisons of the reprogrammed transcriptomes identified major differences in expressed genes and transcriptional regulation, some of which are most likely first stage-specific modules that are necessary for embryonic genome activation. A failure of these transcriptional regulatory pathways would hinder MZT. The inefficiency of mitochondrial protein import might also act as another cause for the developmental failure of iSCNT embryos. Further functional analysis studies on the pathways and genes essential for early embryo development are needed if the efficiency of SCNT and iSCNT is to be improved.

## Methods

### Ethics statement

All the bovine Oocytes and embryos were handled and studies were carried out according to the guidelines of The Inner Mongolia University Animal Care and Use Committee. The bovine ovaries used in this study were collected with permission of the Hohhot slaughterhouse. The animal protocol was approved by The Animal Care and Use Committee of Inner Mongolia University. A small piece of ear tissue of an adult Przewalski’s gazelle was collected in Qinghai Wildlife Garden (Xining) with the permission of Qinghai Forestry Bureau.

### The production of cloned embryos using classic SCNT and reverse nuclear transfer (RNT) protocols

The culture of Przewalski’s gazelle fibroblasts and the in vitro maturation of bovine oocytes were described in our previous reports [55, 56]. The donor cells were transferred to the perivitelline space of enucleated oocytes by the classic SCNT [55, 57] and RNT protocols was described by Meng et al. [39]. Successful enucleation was confirmed by UV illumination of the Hoechst-stained karyoplasts as described above. The enucleated oocytes were allowed to recover for 30 min in an incubator and then activated with ionomycin and CHX treatments. The resulted embryos were cultured for further development.

The reconstructed couplets were electrically fused and then chemically activated by ionomycin and cycloheximide. The activated cloned embryos were cultured in SOFaa in the presence of VPA or TSA at 38.5°C in a humidified atmosphere containing 5% CO_2_. Cleavage and embryo development were observed with a light microscope at 100X. A more detail description of the SCNT and iSCNT protocols can be found in the supplemental materials (Additional file 11).

### Microarray dataset

The Affymetrix Gene Chip Bovine Genome array contains approximately 23,000 transcripts including assemblies from ~19,000 UniGene clusters. The array images were first quantified using Gene Chip Operating Software (GCOS, Affymetrix). The biological replicates of the datasets have high reproducibility.

### Expression pattern analysis

Genes with similar expression patterns are likely to have functional correlations, therefore we performed a cluster analysis of the gene expression patterns using Cluster 3.0 [58] and JavaTreeview [59] software. Expression differences were clustered by the Hierarchical Complete Linkage Clustering method using an uncentered correlation similarity matrix. R packages were used for the Venn diagram and expression analysis of different transcripts.

### Gene ontology analysis

Functional annotation was performed with the Database for Annotation, Visualization and Integrated Discovery (DAVID) Bioinformatics Resource [60]. Gene ontology terms shown in this study summarized all similar sub-terms into an overarching term, and Benjamani-Hochberg adjusted P values were shown for the representative term.

### Statistical analysis

Differences in embryo development between experimental groups were analyzed using Student’s t-test and χ^2^ analysis, SPSS16.0 was used for statistical analysis. P < 0.05 was considered statistically significant.

## Electronic supplementary material

Additional file 1: Table S1: The Cleavage rates, 8-16-cell, morula and blastocyst development of PBNT embryos derived from Oct-4-eGFP transgenic cells. (DOC 34 KB)

Additional file 2: Figure S1: Photomicrographs of the Przewalski’s gazelle-bovine interspecies SCNT embryos and bovine intra-species SCNT embryos cultured *in vitro*. The figures of **A**, **B**, **C**, **D** are different development ages of Przewalski’s gazelle-bovine interspecies SCNT embryos. The figures of **A’**, **B’**, **C’**, **D’** are different development ages of bovine intra-species SCNT embryos. **A**, **A’** are 2–4 cell stage of the embryos (36h post activation). **B**, **B’** are 8–16 cell stage of the embryos (72 h post activation). **C**, **C’** are morula stage of the embryos (120 h post activation). **D**, **D’** are blastocyst stage of the embryos (160 h post activation). Each scale bar represents 100 μm. (DOC 2 MB)

Additional file 3: Table S2: The Spearman correlation coefficients between the profiles of different cell types reflect the degree of change in transcriptomes. (XLS 20 KB)

Additional file 4: Table S3: The expression of 10,000 transcripts in different cell types. BO: bovine oocytes, BBNT: bovine SCNT 8- to 16-cell embryos, PBNT: iSCNT 8- to 16-cell embryos, PC: Przewalski’s gazelle, BC: bovine fibroblast cells. (XLS 1 MB)

Additional file 5: Figure S2: Hierarchical clusters of reprogramming related gene expression profiles. Green indicates the down-and red the up-regulated gene expression. Venn diagram of shared and unique genes among different transcriptomes. (JPEG 3 MB)

Additional file 6: Table S4: The expression of reprogramming related genes in the BBNT(1527) and PBNT(643) embryos. (XLS 2 MB)

Additional file 7: Table S5: The GO annotation of 309 reprogramming related genes that expressed both in BBNT and PBNT embryos. (XLS 42 KB)

Additional file 8: Figure S3: The heatmap profile of the reprogramming related gene that uniquely upexpressed in BBNT embryos and PBNT embryos, respectively. There are respectively 1,218 uniquely up-regulated genes occurred in BBNT embryos and 334 up-regulated genes in PBNT embryos. (PDF 403 KB)

Additional file 9: Table S6: The expression of transcription regulation related genes. One hundred thirty-nine transcripts related to transcription regulation were up-regulated in BBNT embryos; whereas, these genes were down-regulated in PBNT embryos. (XLS 218 KB)

Additional file 10: Figure S4: The expression diversity for basal transcription factors of RNA polymerase in two different embryos. (JPEG 1 MB)

Additional file 11: **The detail description of the SCNT and iSCNT protocols in this study.** (DOC 30 KB)
