# Supplementary material for: Irregular transcriptome reprogramming probably causes thec developmental failure of embryos produced by interspecies somatic cell nuclear transfer between the Przewalski’s gazelle and the bovine
Source: BMC Genomics. 2014 Dec 16;15(1):1113. doi: 10.1186/1471-2164-15-1113 (PMC4378013; doi:10.1186/1471-2164-15-1113)
Supplement: Supplementary file 1 — Additional file 1: Table S1: The Cleavage rates, 8-16-cell, morula and blastocyst development of PBNT embryos derived from Oct-4-eGFP transgenic cells. (DOC 34 KB) [file 12864_2014_6872_MOESM1_ESM.doc]

Table S1. Treatment of Oct-4-eGFP derived PBNT embryos with VPA affect embryo development

| Donor cells | No.embryos cultured | Cleavage (%) | 8-16 cells (%) | Morula (%) | Blastocysts (%) |
| --- | --- | --- | --- | --- | --- |
| Control | 153 | 120(78.4)a | 95(62.1)a | 2(1.3) | 1(0.7) |
| Oct-4-eGFP | 159 | 114(71.7)a | 93(58.5)a | 3(1.9) | 2(1.3) |

In the same column, values with different superscripts differ, p<0.05
