# Supplementary material for: Irregular transcriptome reprogramming probably causes thec developmental failure of embryos produced by interspecies somatic cell nuclear transfer between the Przewalski’s gazelle and the bovine
Source: BMC Genomics. 2014 Dec 16;15(1):1113. doi: 10.1186/1471-2164-15-1113 (PMC4378013; doi:10.1186/1471-2164-15-1113)
Supplement: Supplementary file 2 — Additional file 2: Figure S1: Photomicrographs of the Przewalski’s gazelle-bovine interspecies SCNT embryos and bovine intra-species SCNT embryos cultured in vitro. The figures of A, B, C, D are different development ages of Przewalski’s gazelle-bovine interspecies SCNT embryos. The figures of A’, B’, C’, D’ are different development ages of bovine intra-species SCNT embryos. A, A’ are 2–4 cell stage of the embryos (36h post activation). B, B’ are 8–16 cell stage of the embryos (72 h post activation). C, C’ are morula stage of the embryos (120 h post activation). D, D’ are blastocyst stage of the embryos (160 h post activation). Each scale bar represents 100 μm. (DOC 2 MB) [file 12864_2014_6872_MOESM2_ESM.doc]

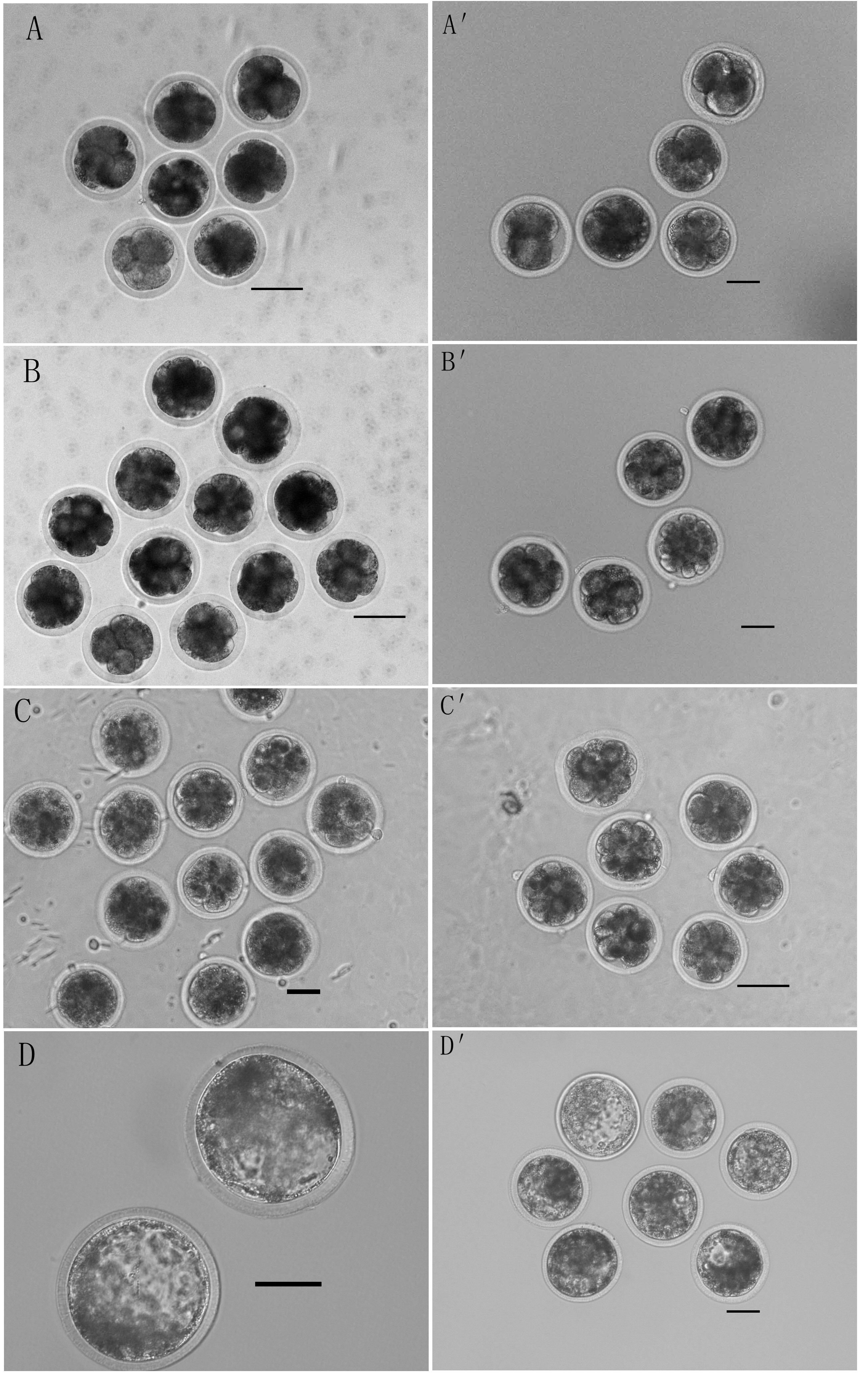


**Figure S1** **Photomicrographs of the Przewalski's gazelle-bovine interspecies SCNT embryos and bovine intra-species SCNT embryos cultured *in vitro*.** The figures of A, B, C, D are different development ages of Przewalski's gazelle-bovine interspecies SCNT embryos. The figures of Aˊ, Bˊ, Cˊ, Dˊ are different development ages of bovine intra-species SCNT embryos. A, Aˊ are 2-4 cell stage of the embryos (36h post activation). B, Bˊ are 8-16 cell stage of the embryos (72h post activation). C, Cˊ are [morula](app:ds:morula) stage of the embryos (120h post activation). D, Dˊ are blastocyst stage of the embryos (160h post activation). Each scale bar represents 100μm.
