# Supplementary figures and images for: Irregular transcriptome reprogramming probably causes thec developmental failure of embryos produced by interspecies somatic cell nuclear transfer between the Przewalski’s gazelle and the bovine
Source: BMC Genomics. 2014 Dec 16;15(1):1113. doi: 10.1186/1471-2164-15-1113 (PMC4378013; doi:10.1186/1471-2164-15-1113)

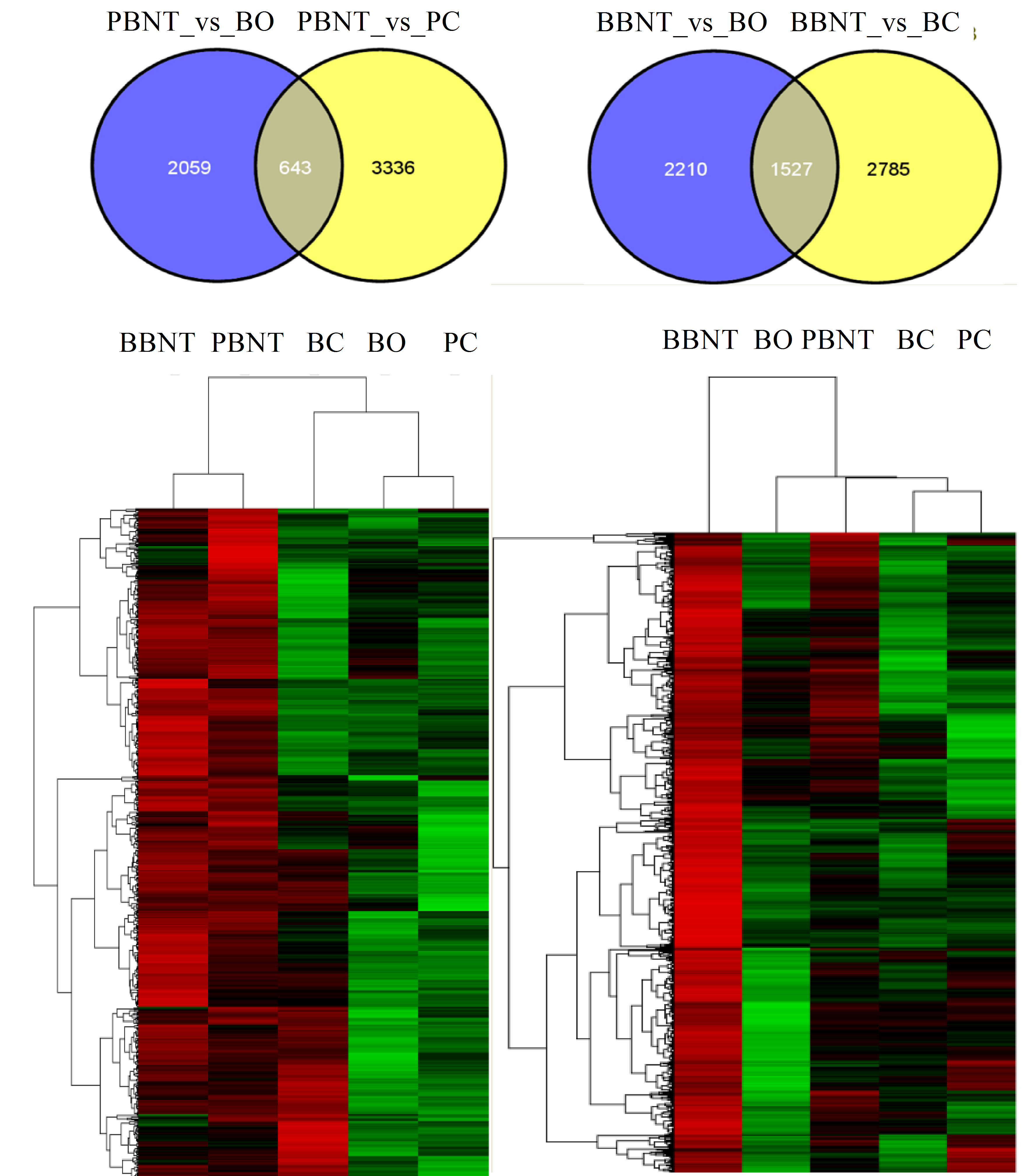

Supplement: Supplementary file 5 — Additional file 5: Figure S2: Hierarchical clusters of reprogramming related gene expression profiles. Green indicates the down-and red the up-regulated gene expression. Venn diagram of shared and unique genes among different transcriptomes. (JPEG 3 MB) [file 12864_2014_6872_MOESM5_ESM.jpeg]

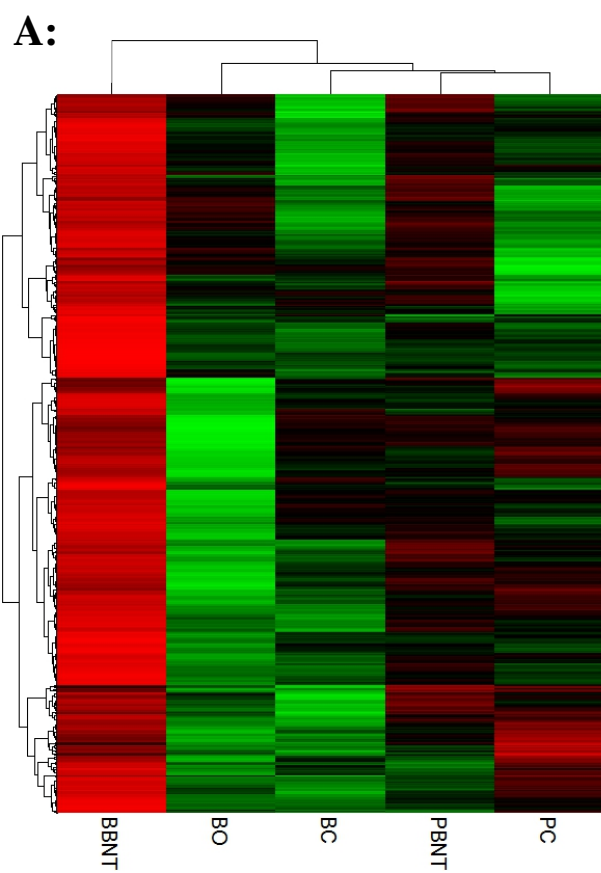

1218 uniquely upregulated genes in BBNT embryos

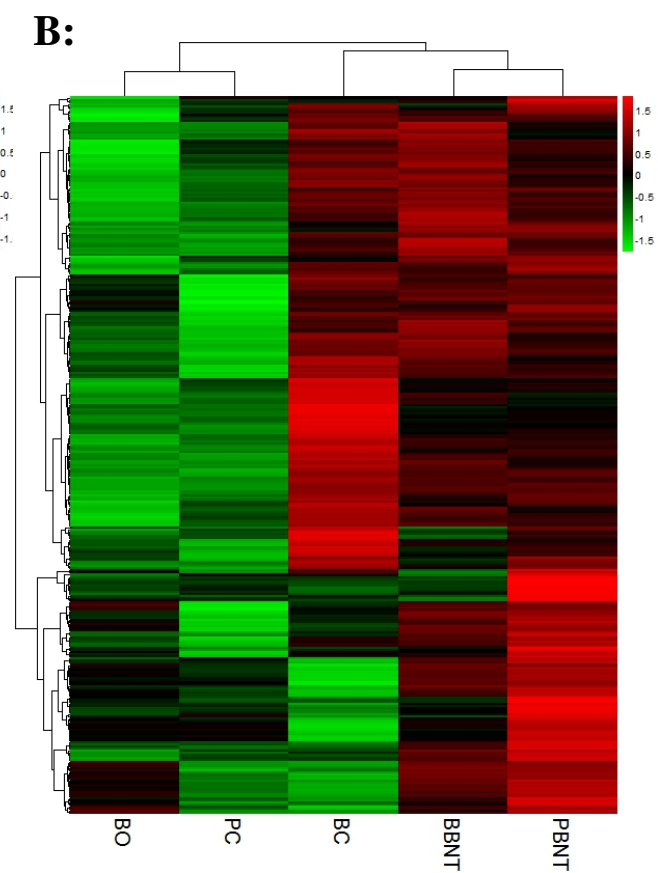

334 uniquely upregulated genes in PBNT embryos

Supplement: Supplementary file 8 — Additional file 8: Figure S3: The heatmap profile of the reprogramming related gene that uniquely upexpressed in BBNT embryos and PBNT embryos, respectively. There are respectively 1,218 uniquely up-regulated genes occurred in BBNT embryos and 334 up-regulated genes in PBNT embryos. (PDF 403 KB) [file 12864_2014_6872_MOESM8_ESM.pdf]

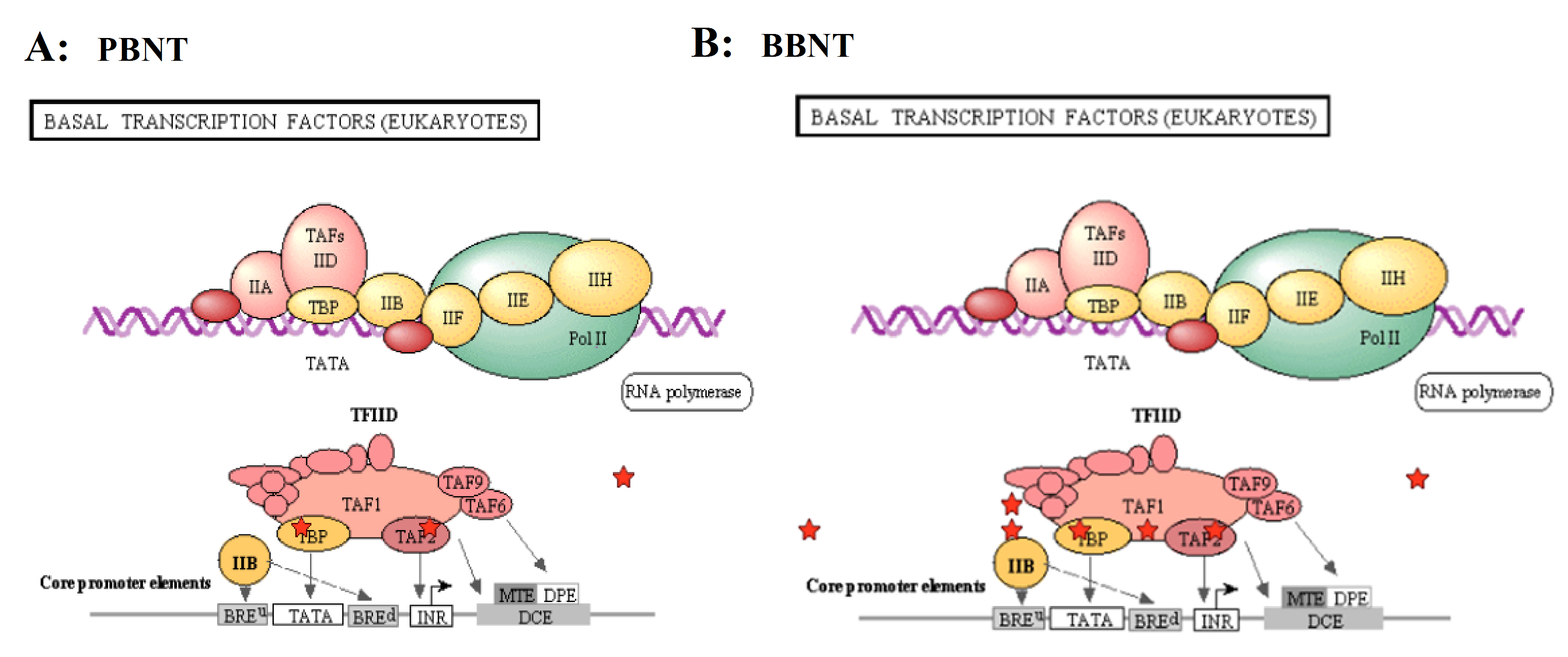

Supplement: Supplementary file 10 — Additional file 10: Figure S4: The expression diversity for basal transcription factors of RNA polymerase in two different embryos. (JPEG 1 MB) [file 12864_2014_6872_MOESM10_ESM.jpeg]
