# Supplementary material for: Irregular transcriptome reprogramming probably causes thec developmental failure of embryos produced by interspecies somatic cell nuclear transfer between the Przewalski’s gazelle and the bovine
Source: BMC Genomics. 2014 Dec 16;15(1):1113. doi: 10.1186/1471-2164-15-1113 (PMC4378013; doi:10.1186/1471-2164-15-1113)
Supplement: Supplementary file 11 — Additional file 11: The detail description of the SCNT and iSCNT protocols in this study. (DOC 30 KB) [file 12864_2014_6872_MOESM11_ESM.doc]

**Additional file 11**

**Chemicals**

Unless otherwise noted, all chemicals used in this study were purchased from Sigma Chemical Co. (St. Louis, MO). Each experiment included at least three replicates.

**Donor Cell Culture and Preparation**

The tissue was separated with 0.1% hyaluronidase (Type-1), washed several times in DMEM/F12 (1:1) (GibcoInc, Grand Island, NY), and cultured in DMEM/F12 supplemented with 10% FBS in 25 cm flasks at 37°C under 5% CO_2_ in air. The cells were passaged when they reached over 80% confluence. Immediately before nuclear transfer, the cells at 80% confluence were dissociated by trypsinization with 0.25% trypsin with EDTA solution (HyClone). Small-size (10-12 µ m) cells with round shape were used as donors[55].

**Bovine Oocyte Maturation in vitro (IVM)**

Maturation of bovine oocytes was as described previously [56].Briefly, bovine cumulus oocyte complexes (COC) were aspirated from 3-8 mm diameter follicles on ovaries obtained from a local slaughterhouse. Only COC with a compact and a homogenous ooplasm were selected. The COC were matured in TCM 199 with Earle’s salts, L-glutamine and sodium bicarbonate (Gibco Inc., Grand Island, NY) supplemented with 10% FBS (HyClone, Logan, Utah, USA), 25 g/ml gentamycin, 0.01U/ml FSH (NIH-FSH-S17), 0.01U/ml LH (USDA-bLH-6) and 1 µM PHE (penicillamine, hypotaurine and epinephrine) in 4-well plates with 0.5 ml medium and 30-50 oocytes/per well at 38.4°C in humidified 5% CO_2_ in air.

**Preparation of Oct-4 transgenic cells**

Briefly, vectors of Oct-4-eGFP and pDS-Red2 were respectively constructed and confected to gazelle fibroblast cells mediated by lipfectamine. A 15 µl lipfectamine LTX and 4 µg DNA plasmids resulted in about 32.0% transfection rate identified by flow cytometry. The pDS-Red2 was as a refer for cell confection and Oct-4 expression was used as a marker for potential ability of embryo development.

**Nuclear transfer, fusion and activation**

The SCNT protocols used in this experiment were as our previous reports [55,57]. Briefly, the selected oocytes were placed into a 30 µL microdrop of M199 medium (Gibco) containing 1% FBS, 7.5 µg·mL^-1^cytochalasin B and 7.5 µg·mL^-1^ Hoechst 33342 covered by mineral oil. The PB1 and the adjacent cytoplast containing oocyte chromosomes were removed by a glass pipette (18-20 mm) under 200 × magnification. The enucleation process was performed under ultraviolet light to ensure removal of the oocyte chromatins. A single cell was placed into the perivitelline space of the enucleated oocytes. The cell-cytoplast complexes were placed in SOFaa containing 0.5% bovine serum albumin (BSA) and allowed to recover for 30 min.

The reconstructed couplets were electrically fused with an ECM 2001 Electro cell Manipulator (BTX), and incubated in SOFaa containing 0.5% BSA for 30 min at 38.5°C in a humidified atmosphere containing 5% CO_2_. The fused embryos were chemically activated by incubation with 5 µM ionomycin for 5 min followed by incubation with 10 µg·mL^-1^cycloheximide (CHX) for 5 h at 38.5°C in a humidified atmosphere containing 5% CO_2_.

**Embryo culture *in vitro***

The activated cloned embryos were cultured in SOFaa plus 0.5% BSA for 48 h and then checked for cleavage. The cleaved embryos were then continued to culture in SOFaa containing 4% FBS and incubated for further 5 days at 38.4°C in a humidified atmosphere containing 5% CO_2_. The medium was replaced with fresh medium every 2 days.

**Reverse nuclear transfer (RNT)**

RNT was described in our previous report [24].Successful enucleation was confirmed by UV illumination of the Hoechst-stained karyoplasts as described above. The enucleated oocytes were allowed to recover for 30 min in an incubator and then activated with ionomycin and CHX treatments. The resulted embryos were cultured for further development.

**Experimental Designs**

***Experiment 1.*** This experiment was designed to investigate whether treatment of cloned embryos with VPA affected the embryo development. The activated PBNT embryos were incubated in the presence of VPA at 0.5, 1.0, 2.0 and 4.0 mM for 24 h, respectively. The cleavage and embryo development were observed.

***Experiment 2.*** In the first experiment, we found VPA at 0.5 mM resulted in higher cleavage and embryo development. This experiment was designed to use 0.5 mM VPA to treat the activated PBNT embryos for 5, 12, 24, and 48 h, respectively to observe the subsequent embryo development.

***Experiment 3.*** The gazelle cells were transfected with Oct-4-eGFP, then the cells were as donors to create PBNT embryos. The embryos were activated and then treated with VPA at 0.5 mM for 24 h to observe the embryo development.

***Experiment 4.*** Combination of OCT-4 transfected cells and TSA treatment of the cloned embryos was conducted in this experiment. The Przewalski's gazelle fibroblast cells and the Oct-4-eGFP transgenic cells were used as donor cells to prepare the PBNT and BBNT cloned embryos. The activated embryos were then incubated in CR1aa medium in the presence of TSA at the concentration of 30 nM. The embryo development was recorded.

***Experiment 5.*** In our previous bovine cloning report, RNT protocol have beneficial effect on the cloned embryo development[24]. The RNT protocol was used in this experiment. The gazelle cells were inserted and fused to intact oocytes, and then the oocyte nuclei were removed 3 h post-fusion as described above. After activation, the embryos were incubated and the development was observed.

***Experiment 6.*** The results from Experiment 1 to Experiment 5, and also data from the published reports showed an extremely lower morula/blasocyst development in iSCNT although a lot of modified protocols were tried. This experiment was to analyze the related gene expressions of the PBNT, BBNT embryos, and the donor cells by Affymetrix gene chip bovine genome array. Total of 1150 bovine oocytes (BO), 309 bovine SCNT 8- to 16-cell embryos (BBNT), 527 iSCNT 8- to 16-cell embryos (PBNT), enough Przewalski's gazelle fibroblast cells (PC) and enough bovine fibroblast cells (BC) were collected.
